# Effects of Priming with Light vs. Heavy Loads on Weightlifting Performance

**DOI:** 10.3390/jfmk10010052

**Published:** 2025-01-30

**Authors:** Theodoros Stavropoulos, Nikolaos Zaras, Georgia-Kassandra Kelekian, Thomas Mpampoulis, Alexandra Avloniti, Athanasios Chatzinikolaou, Gerasimos Terzis

**Affiliations:** 1 Department of Physical Education and Sport Science, School of Physical Education, Sport Science and Occupational Therapy, Democritus University of Thrace, University Campus, 69100 Komotini, Greece; theostav6@phyed.duth.gr (T.S.); alavloni@phyed.duth.gr (A.A.); achatzin@phyed.duth.gr (A.C.); 2 Department of Life Sciences, School of Life and Health Sciences, University of Nicosia, 24005 Nicosia, Cyprus; 3 School of Physical Education and Sport Science, National and Kapodistrian University of Athens, 17237 Athens, Greece; gkkelekian@phed.uoa.gr (G.-K.K.); thompamp@phed.uoa.gr (T.M.); gterzis@phed.uoa.gr (G.T.)

**Keywords:** neuromuscular activation, peak velocity, 1 repetition maximum, Olympic weightlifting

## Abstract

Background/Objectives: The purpose of the study was to investigate the effects of a priming training session with either a light or heavy load snatch and clean pulls on weightlifting performance. Methods: Twelve well-trained weightlifters (seven males and five females) participated in the study. The athletes followed a counterbalanced study design comparing three treatments, including a day of rest (control) and two priming sessions involving two different weightlifting derivatives—the snatch and the clean pulls—which were performed either with 80% of the one-repetition maximum (1-RM) (LP) or with 110% of the 1-RM (HP). Twenty-four hours later, the 1-RM strength test for the snatch and clean and jerk, as well as the barbell kinematic characteristics at 100% of the 1-RM in the snatch and clean and jerk, were measured. The rate of perceived exertion (RPE) was measured following the priming sessions. Results: Performance in snatch remained unchanged following the LP and HP. However, performance in the clean and jerk increased significantly by 3.1% following the HP compared to the control. No significant differences were observed in barbell kinematics. The RPE was significantly higher for HP compared to LP. Conclusions: These results suggest that an HP performed 24 h prior to the 1-RM evaluation in weightlifting may have significantly increased performance in the clean and jerk. These changes may not be explained by barbell kinematics.

## 1. Introduction

Weightlifting is a powerful sport comprising two multi-joint exercises: the snatch and the clean and jerk [1]. Performance in weightlifting depends largely on both biological (i.e., neural activation, lean mass, etc.) [2,3] and biomechanical factors (i.e., barbell kinematic and kinetic analysis) [4,5]. However, the ability of the athletes to produce high amounts of power and apply their maximum force as fast as possible to the barbell remains a factor that significantly contributes to a successful lift. In addition, training in weightlifting is designed according to the principles of periodization, employing a combination of linear, block, and non-linear periodized training schemes, which may vary according to the training period [3,6,7,8]. Nevertheless, the majority of athletes perform a tapering training phase before competitions in an attempt to reduce fatigue accumulated during the long-term training period, mainly by reducing training volume and thereby increasing weightlifting performance for the target competition [9]. Indeed, the results from a study on weightlifters showed that during the tapering phase, athletes performed their last high-intensity training (>85% of 1-RM) approximately 5.9 ± 2.3 days before competitions while the training ceased the day before competition [10]. However, anecdotal data and communications with several coaches and athletes revealed that the day before their competition, athletes may choose to perform a low-volume, moderate to high intensity-training session in an attempt to activate their neuromuscular system for the upcoming competition. This priming training session may include power lifts like the power snatch and power clean and jerk, as well as snatch and clean pulls with loads lower or equal to the first competitive attempt. Therefore, the following question arises here: What type of training can a weightlifter perform 24 h before competition to enhance weightlifting performance?

The delayed potentiation effect or priming has been proven to be an effective training strategy that can be used from 5.5 to 48 h before competition to enhance performance on the day of the competition [11]. Priming is characterized as a short-term training session with low training volume but moderate to high training intensity [12]. The duration of a priming training session does not exceed 30 min while priming may focus on enhancing strength, using either light (ballistic loads <45% of 1-RM) or with heavy loads (>80% of 1-RM), speed and agility with repeated sprints, or even sport-specific exercises [11,12]. The results from studies on soccer have shown that a resistance priming session may have a positive effect on agility [13], self-reported fatigue, the Yo-Yo intermittent aerobic test, and the rate of torque development compared to no priming session [14]. Moreover, a priming resistance training session may significantly enhance strength, vertical jump, and sprinting speed in rugby players compared to no priming or a sprint priming training session [15]. Furthermore, a study of well-trained team-sport athletes showed that a resistance priming session may significantly enhance the rate of force development up to 48 h following the priming session [16]. The possible mechanism underlining these positive changes in performance following a priming training session is similar to the post-activation performance enhancement (PAPE) phenomenon [17], and is mainly driven by the increased muscle fiber sensitivity to calcium ions, which enhances the neuromuscular activation and the rate of force development [6,18], leading to an enhanced muscle contraction and subsequently improving performance [19,20]. Although there is strong evidence regarding the positive effects of priming on performance in several team sports, the effects of priming on weightlifting have not been sufficiently investigated.

In weightlifting, priming or PAPE may be performed using either light or heavy loads. A recent study investigated the effects of a preconditioning clean pull, which a group of female weightlifters performed 2 min before attempting their one repetition maximum (1-RM) in the clean. Preconditioning was performed with either light loads (85% of 1-RM) or heavy loads (120% of the 1-RM) using the clean pulls as a preconditioning exercise. The study showed that performance in the clean increased similarly following the two PAPE protocols (85% = 6.1 ± 3.6% vs. 120% = 4.7 ± 3.1%), although the heavy load precondition elicited significantly greater RPE compared to the lighter precondition. The results of the current study suggest that one set of one, fast velocity clean pull repetition, either with 85% or 120% of the 1-RM clean, may acutely enhance weightlifting performance in the clean [21]. Moreover, an earlier study investigated the effects of a priming training session, including snatch and clean pulls with a low volume and moderate intensity (85% of 1-RM) in a group of 19 young weightlifters, 5 to 6 h prior to a simulated weightlifting competition. The study showed that performance in weightlifting remained unaltered. However, when the athletes were separated into responders (*n* = 6) and non-responders (*n* = 13) the study showed that responders regulated their anxiety levels, and as a consequence, increased their weightlifting performance [22]. According to the authors’ knowledge, no other studies have investigated the effects of a priming session 24 h before the evaluation of 1-RM strength in the snatch and clean and jerk. The results of the current study may be used by coaches and athletes in an attempt to acutely enhance weightlifting performance 24 h before entering either a high intensity training session or a competition.

The purpose of the study was to investigate the effects of a priming session using the snatch and clean pulls performed 24 h before the 1-RM evaluation, either with light or heavy loads on weightlifting performance in a group of well-trained weightlifters. The hypothesis of the study was that both priming sessions would induce greater increases in weightlifting performance compared to a no-priming condition.

## 2. Methods

### 2.1. Experimental Design

The effects of priming with either light or heavy load snatch and clean pulls performed 24 h prior to 1-RM in the snatch and clean and jerk remain unclear. A counterbalanced repeated-measures study design was used. The experimental procedures spanned a duration of 4 weeks. During the first week, all athletes performed the 1-RM strength evaluation in the snatch and clean and jerk following a day of rest and according to their self-selected increase in loads [3]. Then, athletes were randomly assigned to three different experimental conditions: no priming, which was the control condition (C), light load priming (LP), and heavy load priming (HP). During the next three weeks, athletes performed the three experimental conditions in a counterbalanced fashion. To keep all training conditions similar for all athletes, we kept the priming and 1-RM measuring days and hours the same throughout the experimental procedure. At the end of the experimental period, all athletes performed under all three experimental conditions.

### 2.2. Participants

Twelve (N = 12) well-trained weightlifters (7 males: age: 24.6 ± 2.5 years; body height: 177 ± 7 cm; body mass: 87.6 ± 21.5 kg and 5 females: age: 24 ± 2.9 years; body height: 163 ± 4 cm; body mass: 65.8 ± 2.4 kg) with 4.0 ± 2.6 years of training experience participated in the study. Athletes’ maximum strength in the snatch, clean and jerk, and total is presented in Table 1. Before entering the study, athletes fulfilled the following criteria: absence of any cardiovascular conditions and musculoskeletal injuries, engagement in regular training (>5 training sessions per week), a minimum of two years of training experience in weightlifting, and experience competing in the last national championship. In addition, athletes were excluded from the study if they used any illegal substances and/or consumed any energy supplements prior to priming sessions and 1-RM measurements. All procedures were in accordance with the 1975 Declaration of Helsinki as revised in 2000 and were approved by the local ethical committee of the Democritus University of Thrace (project number A.Π.Δ.Π.Θ./Ε.H.Δ.Ε./48170/352).

### 2.3. Training

An experimental procedure was performed approximately 10 weeks prior to a local competition. All priming sessions were performed during the end of the training microcycles. More specifically, the athletes followed three days of training, decreasing volume and intensity in an attempt to reduce muscle fatigue and optimize their 1-RM at the end of the training week. The three days included the snatch and clean and jerk movements as well as weightlifting derivatives like the power snatch and the power clean, the push press and jerk, the snatch and clean pull (from the floor or from boxes), the front and the back squat, and several accessory exercises like the leg extension, the leg curl, the barbell shoulder press, etc. All priming sessions were performed 24 h prior to the 1-RM evaluation in the snatch and the clean and jerk. For the C condition (no priming), athletes refrained from any type of exercise. Priming sessions were performed either with 80% of their 1-RM (LP) or with 110% of their 1-RM (HP) of the snatch and clean and jerk. The percentage loads for each athlete were established during the first week of the experimental procedure when a 1-RM evaluation in the snatch and clean and jerk was performed. The priming training program included two different weightlifting derivatives: the snatch pulls and the clean pulls. The actual priming training program for the LP included 3 sets of 3 repetitions of snatch and clean pulls, while the HP included 3 sets of 2 repetitions of snatch and clean pulls. All repetitions were performed with a 3 s intra-repetition rest. During all priming sessions, athletes were instructed to accelerate the barbell maximally, especially during the second pull, while simultaneously elevating the shoulders and executing a triple extension of the hips, knees, and ankles [1]. Rest intervals were 3 min between sets and 5 min between exercises. Additionally, straps were provided during the repetitions to prevent finger strain caused by the heavy loads during the priming sessions. At the end of the training session, the athletes’ rating of their perceived exertion (RPE) was recorded on a scale from 6 to 20 [21].

### 2.4. Maximum Strength in Snatch and Clean and Jerk

All 1-RM strength measurements were performed in the training facilities of the athletes. During the 1st week, 1-RM strength was measured according to a self-selected progression in barbell loading. During the next three 1-RM evaluations, athletes followed a consistent progression in barbell loading, which was the same for the snatch and the clean and jerk: 2–3 sets with an empty barbell for 5–6 repetitions, 2 sets with 50% of their 1-RM for 3 repetitions, 2 sets with the 65% of their 1-RM for 2 repetitions, 1 set with 75% of their 1-RM for 2 repetitions, 1 set with 85% of their 1-RM for 2 repetitions, 1 set with 95% of their 1-RM for 1 repetition, and 3 sets of 1 repetition each with 100% of their 1-RM. When an athlete was able to lift 100% of his/her personal best, then a 2.5% increase in load was given for the second and third attempts. During all measurements, a certified weightlifting coach was present to evaluate the correct execution of the lifts according to the rules of the International Weightlifting Federation (IWF). The best performance in the snatch in the clean and jerk, as well as in the total, was used for the statistical analysis.

### 2.5. Barbell Kinematic Characteristics

Barbell kinematic characteristics were evaluated during 100% of the athletes’ 1-RM in the snatch and the clean and the jerk. More specifically, a linear position transducer (Vitruve LPT, sampling frequency: 100 Hz, SPEED4LIFTS S.L., Calle Rio Segre, Mostoles, Madrid) was attached to the barbell using a cord, and measured the barbell kinematics during the pulling phase (first and second pull) of the snatch and the clean as well as during the pushing phase of the jerk. The cord was secured externally to the barbells’ weight-loading sleeve and easily removed to add an extra load to the barbell. The linear transducer was placed directly under the barbell during the initiation of the first pull in an attempt to minimize the horizontal barbell movement during the movement [23]. The linear position transducer measured the mean propulsive velocity (MV), the peak velocity (PV), and the vertical displacement of the barbell (VD). In order to familiarize the athletes with the presence of the encoder, the linear transducer was placed on the weight-loading sleeve during the first week of measurement for all loads, starting from the warm-up with an empty barbell to the 100% of 1-RM in both snatch and clean and jerk.

### 2.6. Statistical Analysis

The sample size was determined using a post hoc sample power analysis. The analysis showed power of 0.979 for 12 athletes. All data are presented as means and standard deviations. Following the Kolmogorov–Smirnov test, all data were normally distributed. A one-way repeated measures analysis of variance was used to investigate the differences between C, LP, and HP conditions. Eta squared (η^2^) was calculated for the effect size for differences between the treatment conditions. In case of a significant main effect, the Bonferroni adjustment was used to compare the differences between the three priming conditions. Differences in the RPE values between LP and HP were compared with a paired sample *t*-test. Moreover, the percentage change comparisons between the LP and HP with the C condition were also performed with a paired sample *t*-test. Statistical significance was set at *p* ≤ 0.05.

## 3. Results

All athletes completed all measurements without injuries. A significant difference was found for RPE between LP and HP sessions (t = −4.005, *p* = 0.002). Specifically, RPE was significantly higher following the HP compared to LP (HP: 10.8 ± 2.7, vs. LP: 8.9 ± 2.3, *p* = 0.002). Table 2 presents the results of weightlifting performance following all priming sessions. No significant difference was found in snatch between the three different priming sessions (Wilk’s Lambda = 0.907, F_(10,2)_ = 0.510, η^2^ = 0.093, *p* = 0.615). However, a significant main effect was found for the clean and jerk (Wilk’s Lambda = 0.428, F_(10,2)_ = 6675, η^2^ = 0.572, *p* = 0.014). Pairwise comparisons indicated that performance in the clean and jerk was significantly increased only after the HP session compared to the C (*p* = 0.008). Repeated-measures ANOVA revealed a statistical trend in total weightlifting performance (Wilk’s Lambda = 0.598, F_(10,2)_ = 3355, η^2^ = 0.402, *p* = 0.077). This tendency seemed to arise from the pairwise comparisons between the HP and C conditions (*p* = 0.065).

Table 3 presents the results of the barbell kinematic characteristics. No significant differences were found in MV, PV, and VD following all three priming conditions.

Table 4 presents the individual results from the priming sessions in weightlifting performance as well as the percentage differences between the LP and HP with the C condition. The percentage difference between HP and C was significantly higher for the clean and jerk compared to the percentage difference between LP and C (t = −2.538, *p* = 0.028). Additionally, the percentage difference between the HP and C was significantly higher for total compared to the percentage difference between the LP and C (t = −2.113, *p* = 0.05). However, no significant difference was found between the percentage difference in the HP and C compared to the percentage difference in the LP and C for snatch (t = −0.850, *p* = 0.413).

## 4. Discussion

The purpose of the study was to investigate the effects of LP vs. HP performed 24 h prior to the 1-RM evaluations of the snatch and clean and jerk in a group of well-trained weightlifters. The hypothesis was that both LP and HP would enhance weightlifting performance. The main finding of the study was that HP significantly increased the clean and jerk performance and showed a trend toward increasing performance in total. In addition, both LP and HP sessions induced a positive effect on weightlifting performance compared to the no priming condition. However, these positive effects on weightlifting performance were not accompanied by changes in the kinematic characteristics of the barbell during the 100% of 1-RM. MV, PV, and VD remained unchanged following all three priming conditions. Interestingly, the HP session resulted in a significantly greater percentage increase in the clean and jerk and a tendency for an increase in the total compared to the LP session. Furthermore, nine out of twelve weightlifters (five males and four females) increased their clean and jerk performance following the HP, suggesting that a robust neuromuscular activation occurred as a result of the HP session. In contrast, only four athletes (two males and two females) increased their snatch performance following HP. From a biomechanical perspective, the snatch is a more technical weightlifting movement which requires power–speed development in a single continuous movement [1,4]. Unlike the snatch, the clean and jerk is a relatively slower and more dynamic weightlifting movement [1]. Therefore, the lack of significant increases in the snatch following the priming sessions may be partially explained by the fact that it is a more technically complex movement, which has higher velocity demands compared to the clean and jerk. These results suggest that coaches may incorporate an HP training session with loads of 110% of 1-RM in the snatch and the clean and jerk, 24 h prior to 1-RM evaluation or a competition to maintain snatch performance, significantly increase clean and jerk performance, and enhance performance in total. However, these changes may not be explained by the barbell’s kinematic characteristics.

Resistance priming has been shown to significantly enhance neuromuscular activation and the rate of force development, leading to enhanced muscle contraction activation and, subsequently, increased sports performance [11]. In weightlifting, priming with heavy loads induced a significant increase in the clean and jerk by 3.1% compared to the C condition. During the pre-competition period, weightlifters frequently perform 1-RM evaluations in the snatch and the clean and jerk. Consequently, priming with heavy loads, which are adjacent to the force factor of the force–velocity curve [24], may induce a stronger neuromuscular activation compared to lighter loads or absence from training. The HP training session consisted of the snatch and clean pulls at 110% of 1-RM in the respective exercises, while athletes were instructed to achieve their maximum intentional movement velocity, particularly during the second pull. A previous meta-analysis showed that this instruction may increase the recruitment of fast motor units, leading to greater increases in the rate of force development and, as a consequence, an enhanced presence of the positive effects of the PAPE phenomenon [25]. Similarly, a study on power athletes (track and field throwers) demonstrated that 2 weeks of tapering with heavy loads (85% of 1-RM) and with maximum intentional movement velocity during training may significantly increase strength, rate of force development, and power compared to lighter loads (30% of 1-RM) [26]. Moreover, an earlier study on 19 young weightlifters showed that a priming training session with 85% of 1-RM, 5.5–6 h before a simulated weightlifting contest, resulted in no significant difference in weightlifting performance. However, the responded weightlifters of this study experienced increases of 5.8 kg in the snatch and 6.2 kg in the clean and jerk compared to the non-responders. In addition, the priming session appeared to regulate the high levels of anxiety that the responders experienced during the day of the competition [22]. From these results, we can only hypothesize that LP may not provide a strong neuromuscular stimulus to produce detectible performance improvements 24 h following the priming session in weightlifters. Therefore, when the priming session is planned 24 h prior to 1-RM evaluation or competition, then a HP session should be preferred. In contrast, if the priming session is scheduled 5.5–6 h before the 1-RM evaluation or competition then, perhaps, a LP session may be more suitable [22].

One interesting finding of the study was that performance in total tended to increase following the HP compared to C (*p* = 0.077). This 2.21% increase should not be overlooked unnoticed by coaches and athletes. Observing the results from the last IWF World Cup held in Thailand (https://tawa.or.th/2024iwfworldcup/en/, accessed on 20 April 2024), it becomes clear that the difference between the 1st and the 2nd or the 2nd and the 3rd podium positions were lower than 2.21% in several body mass categories. Therefore, in real-world conditions, even the smallest increase in total performance may determine the win or the loss in a competition [26]. Moreover, the percentage increases in the clean and jerk and in the total compared to C, were significantly greater following HP compared to LP for the majority of the athletes (nine out of twelve). From a psychological point of view, lifting 10% more weight from the official weight record in the snatch and the clean and jerk during the HP may acutely enhance the self-confidence of the athletes for next-day 1-RM measurements, as previously described [22]. In addition, several physiological parameters like the recruitment of faster and bigger motor units, the high amount of muscle mass activated, and the increase in the rate of force development [16,25] may induce a greater neuromuscular activation compared to the LP. These results reinforce the above hypothesis that HP induced a stronger neuromuscular activation compared to LP. However, these positive effects from HP were accompanied by a higher RPE compared to LP. A previous study of well-trained female weightlifters showed that high-load preconditioning (120% of clean 1-RM) may induce significantly greater RPE values compared to lighter preconditioning (85% of clean 1-RM) [21]. Therefore, coaches should be cautious when using HP sessions, while at the same time, the priming sessions should be individualized according to weightlifters’ conditioning levels. The main goal of tapering is the reduction in fatigue accumulated from long-term training [9]. A recent study showed that during tapering, weightlifters refrain from training 24 h before competition to minimize fatigue [10]. Consequently, increased RPE 24 h prior to 1-RM strength evaluation or a competition could potentially undermine weightlifting performance. For that reason, it is recommended that all priming sessions should first be tested during training before being applied prior to official competitions.

In an attempt to explain the possible effects of priming on weightlifting performance, a linear position transducer was placed on the weight-loading sleeve of the barbell during the athletes’ 1-RM attempts. The results from the linear position transducer revealed no significant differences in MV, PV, and VD in the snatch and the clean and jerk. These findings suggest that changes in weightlifting performance following either LP or HP training sessions may not be explained by using barbell kinematics. For a successful lift, the vertical barbell velocity (both MV and PV) should be progressively increased until the end of the second pull [27], while the velocity during the second pull should be higher compared to the first pull in both the snatch and the clean [27,28]. Therefore, barbell velocity is an integral component of the technique during the snatch and the clean, while it is crucial for a successful lift. However, the PV values in the snatch and clean seemed to be similar to the values of the snatch and the power clean from previous studies [1,6,27,29,30,31,32]. A previous study on seven international-level weightlifters investigated the acute effects of potentiation from heavy sets on a lighter set of dynamic mid-thigh pulls. The study showed an increased PV when heavy sets of dynamic mid-thigh pulls preceded a lighter set [33]. It seems that the barbell’s vertical velocity may acutely increase as a result of a short-term adaptation (PAPE), but it does not appear to be affected by a priming training session. In addition to the barbell velocity, the VD remained unaltered following the priming training sessions, which underpin the effort of all athletes to pull the 1-RM loads as high as possible during all 1-RM evaluations. Previous studies have shown that vertical displacement is similar in both successful and unsuccessful lifts [27,28]. The absence of significant differences in barbell kinematics between priming conditions may partially be explained by the full competitive technique used by athletes (including the transition phase) and the possible effort of the athletes to concentrate on lifting the load at 100% of their 1-RM, and not on maximizing the velocity of movement [34]. In addition, the barbell moves independently from the athlete; hence, the barbell kinematical characteristics may provide different information in relation to the system (athlete–barbell), especially in 1-RM loads [34]. Moreover, a study on seven high-level male weightlifters showed that during repeated maximum lifts, the kinematical characteristics of the barbell remained unchanged, although the direction of the forces applied to the barbell seemed to be decisive for the successful lift [27]. Unfortunately, we were not able to measure the system’s kinetic parameters. Therefore, barbell kinematics alone may not be able to explain the changes in performance following the priming sessions. However, according to the author’s knowledge, this is the first study to investigate changes in barbell kinematics following LP or HP training sessions. Consequently, further studies are needed to investigate the role of barbell kinematics following short-term training sessions in weightlifters.

This study has some limitations. The number of athletes was relatively small; however, recruiting athletes and minimizing the interference with their daily training programs can be challenging. Moreover, although linear position transducers have several benefits, they can only measure the kinematic characteristics of the barbell and not the kinetic variables which can be assessed using force platforms. Additionally, the calculation of the horizontal barbell movement needs basic trigonometry; it was not possible to perform this in the current study [23]. No EMG data were collected following the priming sessions, which might have provided greater insights into the underlying mechanisms of the observed results. Although athletes followed the same training program during the experimental procedure, external factors like sleep, nutrition, or psychological state were not evaluated. Furthermore, under real-world conditions it was impossible to control for the Hawthorne effect and blind athletes during the treatments [35]. Future studies should focus on the investigation of priming sessions involving different weightlifting derivatives or the full snatch and clean and jerk techniques, incorporating different resistance loads and assessing the effects at varying time intervals (hours or days) before competitions.

## 5. Conclusions

In conclusion, a priming training session performed with heavy loads (snatch and clean pulls at 110% of 1-RM) 24 h prior to the 1-RM strength evaluation in the snatch and the clean and jerk may significantly increase 1-RM strength in clean and jerk and, likely, performance in total. However, these positive effects on performance will induce significant increases in RPE; therefore, coaches should be cautious. These positive changes in weightlifting performance may not be reflected by changes in barbell kinematics. Priming with lighter loads (i.e., 80% of 1-RM) may not induce a negative impact on weightlifting performance, although a heavier priming session may induce greater neuromuscular activation.

## 6. Practical Implications

One major aim of tapering is to increase performance in certain competitions [10]. In addition to tapering, priming training sessions performed 24 h prior to the competition or 1-RM evaluation may contribute to enhanced weightlifting performance. Coaches and weightlifters may utilize an HP training session with snatch and clean pull derivatives at 110% of the 1-RM of the snatch and the clean and jerk 24 h prior to 1-RM evaluation or a competition to significantly enhance clean and jerk performance and, partially, total performance. Although tapering aims to reduce RPE prior to competitions, coaches should be aware that an HP session will induce a higher RPE compared to LP or the absence of training. Therefore, coaches should individualize the priming sessions, and any new priming strategy should be first implemented under training conditions before it is used prior to competitions. Moreover, an LP training session will not induce a negative impact on weightlifting performance, although an HP session will induce greater neuromuscular activation. Coaches should regularly evaluate barbell kinematical characteristics during training in various percentages of 1-RM as a tool to monitor training-induced adaptations. However, when 1-RM attempts are performed, then perhaps barbell kinematics may not reflect the changes in performance following short-term training interventions.

## Figures and Tables

**Table 1:** Maximum strength in snatch, clean and jerk and total for the athletes participated in the study.

	Body Mass Category (kg)	Snatch (kg)	Clean and Jerk (kg)	Total (kg)
Female 1	64	47.5	67.5	115
Female 2	59	45	60	105
Female 3	64	40	57.5	97.5
Female 4	71	32.5	45	77.5
Female 5	64	65	75	140
Male 1	67	95	115	210
Male 2	96	95	120	215
Male 3	+109	82.5	120	202.5
Male 4	102	110	128	238
Male 5	67	65	87.5	152.5
Male 6	61	82.5	105	187.5
Male 7	96	95	115	210

**Table 2:** The results in weightlifting performance expressed as mean and standard deviations following the three different priming strategies.

	Control (N = 12)	Light Priming (N = 12)	Heavy Priming (N = 12)	*p*
Snatch (kg)	71.3 ± 25.6	71.3 ± 25.8	71.9 ± 26.0	0.615
Clean and Jerk (kg)	91.3 ± 29.3	92.1 ± 28.8	94.1 ± 30.4 *	0.014
Total (kg)	162.5 ± 54.6	163.4 ± 54.1	166.0 ± 56.2	0.077

* = significant difference between heavy priming and control.

**Table 3:** Changes in barbell kinematics following the control and light and heavy priming sessions during the pulling phase of the snatch and the clean and jerk, and during the pushing phase of jerk.

	Control (N = 12)	Light Priming (N = 12)	Heavy Priming (N = 12)	F	η^2^	*p*
**Snatch**						
MV100% of 1-RM (m·sec^−1^)	1.06 ± 0.11	1.05 ± 0.12	1.04 ± 0.12	0.385	0.088	0.693
PV100% of 1-RM (m·sec^−1^)	2.00 ± 0.17	1.99 ± 0.13	1.92 ± 0.36	0.206	0.049	0.818
VD100% of 1-RM (cm)	121.6 ± 9.7	121.8 ± 9.3	122.5 ± 9.8	0.190	0.045	0.830
**Clean**						
MV100% of 1-RM (m·sec^−1^)	0.86 ± 0.15	0.87 ± 0.12	0.86 ± 0.13	0.236	0.050	0.795
PV100% of 1-RM (m·sec^−1^)	1.69 ± 0.15	1.63 ± 0.11	1.68 ± 0.16	1.738	0.279	0.230
VD100% of 1-RM (cm)	100.3 ± 7.9	99.2 ± 8.3	98.2 ± 8.0	1.039	0.188	0.393
**Jerk**						
MV100% of 1-RM (m·sec^−1^)	0.92 ± 0.10	0.93 ± 0.08	0.95 ± 0.10	0.877	0.163	0.449
PV100% of 1-RM (m·sec^−1^)	1.61 ± 0.15	1.60 ± 0.14	1.61 ± 0.14	0.431	0.087	0.663
VD100% of 1-RM (cm)	51.2 ± 7.2	51.4 ± 5.8	50.9 ± 7.4	0.064	0.014	0.938

MV = mean propulsive velocity, PV = peak velocity, VD = barbell vertical displacement, and 1-RM = one repetition maximum.

**Table 4:** Individual changes in weightlifting performance following the light and heavy priming sessions compared to control (no priming).

	Increased Performance (n)	Decreased Performance (n)	Maintained Performance (n)	% Change vs. Control
**Snatch**				
Light Priming (N = 12)	3 athletes (1 male, 2 females)	4 athletes (2 males, 2 females)	5 athletes (4 males, 1 female)	0.29 ± 4.04
Heavy Priming (N = 12)	4 athletes (2 males, 2 females)	3 athletes (2 males, 1 female)	5 athletes (3 males, 2 females)	1.14 ± 3.67
**Clean and Jerk**				
Light Priming (N = 12)	5 athletes (2 males, 3 females)	1 (1 male)	6 athletes (4 males, 2 females)	1.29 ± 2.85
Heavy Priming (N = 12)	9 athletes (5 males, 4 females)	0 athletes	3 athletes (2 males, 1 female)	3.11 ± 2.25 *
**Total**				
Light Priming (N = 12)	5 athletes (3 males, 2 females)	3 athletes (3 males)	4 athletes (1 male, 3 females)	0.83 ± 2.39
Heavy Priming (N = 12)	7 athletes (4 males, 3 females)	0 athletes	5 athletes (3 male, 2 females)	2.21 ± 2.48 *

* = significant difference between the percentage differences in light and heavy priming sessions.

## Data Availability

The data from this article will be made available by the authors on reasonable request.
